# Effects of local biotic neighbors and habitat heterogeneity on seedling survival in a spruce‐fir valley forest, northeastern China

**DOI:** 10.1002/ece3.3030

**Published:** 2017-05-18

**Authors:** Xucai Pu, Yu Zhu, Guangze Jin

**Affiliations:** ^1^ Center for Ecological ResearchNortheast Forestry UniversityHarbinChina

**Keywords:** generalized linear mixed model, negative density dependence, niche partitioning, seedling dynamics, species coexistence

## Abstract

Seedlings are vulnerable to many biotic and abiotic agents, and studying seedling dynamics helps understand mechanisms of species coexistence. In this study, the relative importance of biotic neighbors and habitat heterogeneity to seedling survival was examined by generalized linear mixed models for 33 species in a spruce‐fir valley forest in northeastern China. The results showed that the relative importance of these factors varied with species and functional groups. Conspecific negative density dependence (CNDD) was important to the survival of *Abies nephrolepis* and *Picea koraiensis* seedling, whereas phylogenetic negative density dependence (PNDD) was critical to *Pinus koraiensis* and *Betula platyphylla*, as well as functional groups of tree, deciduous, and shade‐intolerant seedlings. For shrubs and *Acer ukurunduense*, habitat heterogeneity was significant. Despite of the significance of CNDD, PNDD, and habitat heterogeneity on seedling survival, large proportions of the total variance were not accounted for by the studied variables, suggesting the needs to examine the influences of other factors such as pests, diseases, herbivores, forest structure, species functional traits, and microclimatic conditions on seedling survival in the future.

## 1 INTRODUCTION

Understanding the mechanisms of the maintenance of diversity in plant communities and their relative importance remains a major challenge for ecologists. A number of theories have sought to explain the mechanisms of species coexistence; negative density dependence (NDD) and niche partitioning are two of the widely discussed mechanisms underlying the maintenance of species diversity (Chesson, 2000; Wright, 2002). Recent studies have provided strong evidence that an individual plant will have a low probability of survival when surrounded by a high density of conspecific neighbors or when located close to conspecific trees, supporting the effects of NDD (Chen et al., 2010; Comita, Muller‐Landau, Aguilar, & Hubbell, 2010; Janzen, 1970). On the other hand, species performance is habitat‐specific, indicating that niche partitioning plays an important role in maintaining species coexistence (Metz, 2012; Zhang, Mi, Shao, & Ma, 2011; Zhu, Mi, Ren, & Ma, 2010). An increasing number of studies have shown that NDD and niche partitioning are not mutually exclusive but rather foster the coexistence of species simultaneously (Bai et al., 2012; Chen et al., 2010; Lu et al., 2015; Queenborough, Burslem, Garwood, & Valencia, 2009).

Although conspecific negative density dependence (CNDD) is a very essential process maintaining species coexistence (Comita et al., 2014; Jansen et al., 2014; Piao, Comita, Jin, & Kim, 2013), grouping neighbors into conspecific and heterospecific can be overly simplistic (Metz, Sousa, & Renato, 2010; Webb, Gilbert, & Donoghue, 2006; Wu et al., 2016). Some recent studies have found that neighbors that are more closely phylogenetically related to a focal individual have a stronger negative impact on focal plant survival, a phenomenon called phylogenetic negative density dependence (PNDD; Liu et al., 2012; Paine et al., 2012; Zhu, Comita, Hubbell, Ma, & Shefferson, 2015), which can be seen as an extension of CNDD across evolutionary distance between two neighboring species (Liu et al., 2012; Metz et al., 2010; Webb et al., 2006).

Species of different attributes respond differently to intrinsic and extrinsic factors (Comita & Hubbell, 2009). Growth form may be a key trait in determining the patterns of species survival. Compared with shrubs, trees have larger statures and crowns and can thus produce more seeds and gather more resources; hence trees are often thought to have stronger NDD effects than shrubs (Terborgh, Zhu, Alvarez‐Loayza, & Cornejo‐Valverde, 2014). Some studies have found that leaf habit is related to species survival. Evergreen species can invest a higher proportion of resources to defensive compounds (such as lignin and tannin) than deciduous species; thus deciduous species are usually assumed to suffer more damages from extrinsic factors (Coley & Barone, 1996). Shade tolerance also is an important determinant of species’ reactions to their local biotic neighbors because shade‐tolerant species are less susceptible to enemy (such as herbivores and pathogens) attack than light‐demanding species, so can survive and recover from NDD effects more easily (Coley & Barone, 1996; Kitajima & Poorter, 2010; Kobe, 1997; Myers & Kitajima, 2007). Within a species, tree size could influence the effects of biotic and abiotic variables on survival; smaller individuals may be more sensitive to neighbor density due to asymmetric competition with taller individuals (Uriarte, Condit, Canham, & Hubbell, 2004). Species responses may also vary with dispersal modes, seed production, and individual abundance (Clark, Macklin, & Wood, 1998; Johnson, Beaulieu, Bever, & Clay, 2012).

In tree communities, the transition from seedling to sapling is generally a bottleneck in tree establishment (Queenborough, Burslem, Garwood, & Valencia, 2007). Compared with saplings, seedlings may suffer more from NDD effects and abiotic factors (Wright, 2002); thus, it is essential to explore the process of maintaining diversity and species coexistence in the early stages of plant communities.

Few studies have been undertaken to examine the relative importance of biotic neighbors by consideration of both phylogenetic relatedness and habitat variables in studying seedling survival patterns. In this study, we examined the relative importance of biotic neighbors and habitat variables in seedling survival over 4 years using a dataset of 6,256 seedlings and 33 species in a spruce‐fir valley forest, northeastern China. Our specific questions are as follows:

1. How do biotic neighbors and habitat variables affect seedling survival? Is the CNDD more important than PNDD and habitat variables in seedling survival?
2. Does the importance of biotic neighbors and habitats differ with growth forms, leaf habits, and shade tolerance? More specially, is CNDD or PNDD higher for trees than for shrubs, for deciduous trees than for evergreen trees, and for shade‐intolerant trees than for shade‐tolerant trees?

## 2 MATERIALS AND METHODS

### 2.1 Study site

**Figure 1:**
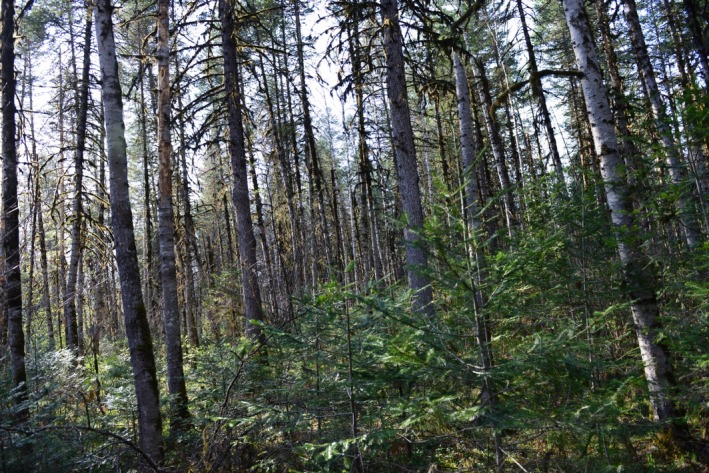
An view of a spruce‐fir valley forest in Liangshui, Heilongjiang province, northeastern China. The species composition primarily includes *Abies nephrolepis*,* Picea koraiensis*,* Acer ukurunduense*,* Pinus koraiensis*,* Betula costata*,* and Larix gmelinii*

**Figure 2:**
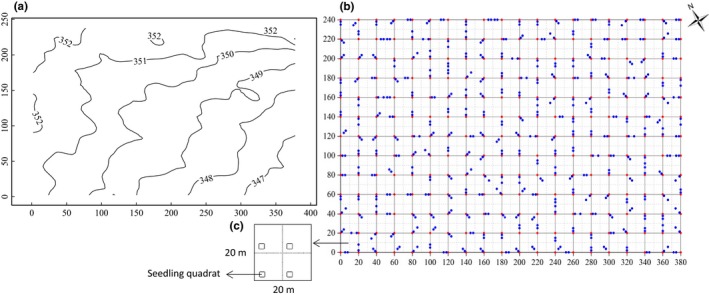
The contour map of the studied spruce‐fir valley forest, northeastern China (a); red dots represent for the every grid points of 20 × 20 m plot where the soil is sampled and blue dots for locations of the two additional soil sample points (b); a panel to show four seedling quadrats inside the 20 × 20 m plot (c)

The study was conducted in a spruce‐fir valley forest in the Liangshui National Nature Reserve (128°53′20″ E, 47°10′50″N), northeastern China (Figure 1). The elevation is 346–352 m (Figure 2). The mean annual temperature is −0.3°C with a mean daily maximum temperature of 7.5°C and minimum temperature of −6.6°C. The mean annual precipitation is 676 mm with a total evaporation of 805 mm. The soil is dark‐brown forest soil and 60 to −80 cm in depth [by Chinese classification, see Shi and Jin (2016)].

In 2006, a 9.12‐ha (380 × 240 m) plot was established in the spruce‐fir valley forest, and censuses were carried out every 5 years. All woody stems with a diameter at breast height (DBH) ≥1 cm were tagged, identified by species, measured, and mapped (Figure 2).

### 2.2 Seedling census

In 2007, a total of 912 seedling quadrats (2 × 2 m) were established in the west corner of each 10 × 10 m subplot within the 9.12‐ha plot (Figure 2). All live woody seedlings ≥30 cm tall and <1 cm dbh were tagged, measured, and identified to species within each seedling plot every 2 years, and after 2011, we added small seedlings ≥10 cm and <30 cm tall to the census.

### 2.3 Biotic neighborhood variables

Four biotic variables for each focal seedling individual were calculated to quantify local neighbor effects, two for seedling neighbors (conspecific neighbor densities and heterospecific neighbor densities) of trees and shrubs using 2011 data, and two for adult tree (all woody stems ≥ 1 cm dbh) neighbors (conspecific neighbor density index and average phylogenetic dissimilarity index) using 2016 data. For seedling neighbors, conspecific seedling neighbor density (*S*_con_) and heterospecific seedling neighbor density (*S*_het_) were calculated. The seedlings on the 10 m edge of the 9.12‐ha study plot were excluded from the calculations, which included 120 of 912 total focal seedling quadrats. For adult tree neighbors, both tree size and phylogenetic factors were considered in the calculations of the conspecific neighbor density index (*A*_con_) and the average phylogenetic dissimilarity index of the heterospecific neighbors (*A*_phylo_; Chen et al., 2016). Four biotic variables for each focal seedling individual were calculated as follows:

$$S_{\mathrm{con}}=N_{\mathrm{con}}$$

$$S_{\mathrm{het}}=N_{\mathrm{het}}$$

$$A_{\mathrm{con}}=\sum_{i}^{N}{\text{BA}}_{\mathrm{con}}^{i}\times W_{i}$$

$$A_{\mathrm{phylo}}=\sum_{i}^{N}\left({\text{PD}}_{i}\times {\text{BA}}_{\mathrm{het}}^{i}\times W_{i}\right)/n$$

$$W_{i}=\text{Exp}\left[-\frac{1}{2}{\left(\frac{{\mathrm{Dist}}_{i}}{R}\right)}^{2}\right]$$

where *S*_con_ is the conspecific seedling neighbor density index; *S*_het_ is the heterospecific seedling neighbor density index; *A*_con_ is the conspecific adult tree density index; *A*_phylo_ is the heterospecific adult tree phylogenetic dissimilarity index; *N*_con_ is the total numbers of conspecific neighbors within the focal individual seedling quadrat (2 × 2 m); *N*_het_ is the total numbers of heterospecific neighbors within the focal individual seedling quadrat (2 × 2 m); BA_con_i is the conspecific neighbor basal area; BA_het_i is the heterospecific neighbor basal area; *i* is the neighboring adult tree; *W*_*i*_ is the Gaussian weight function, which represents the influence weight of neighbor adult tree *i* to the focal seedling; PD_*i*_ is a pairwise phylogenetic distance between focal seedling and its adult tree neighbors; Dist_*i*_ is the spatial distance between focal seedling and the *i*th adult tree neighbor; *R* is the neighborhood influence radius (Table S1); and *n* is the total number of heterospecific neighbors within the focal individual neighborhood influence radius.

**Figure 3:**
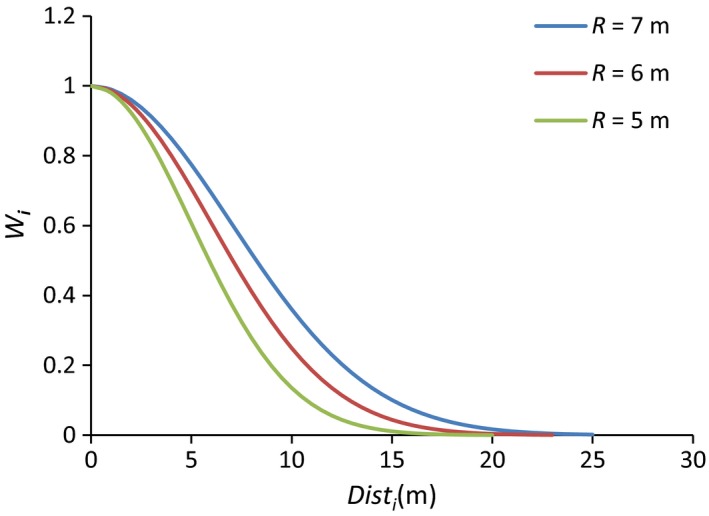
The Gaussian density neighbor kernel with different neighbor influence radius (*R*). *R* = 5 m represents the focal seedling species are *Betula costata*,* Betula platyphylla*,* Juglans mandshurica,* and their nearest phylogenetic‐related species; *R* = 6 m represents the focal seedling species are *Abies nephrolepis*,* Picea koraiensis*,* Pinus koraiensis,* and their nearest phylogenetic‐related species; *R* = 7 m represents the focal seedling species are *Fraxinus mandschurica* and their nearest phylogenetic‐related species

The Gaussian weight function was used instead of the former inverse distance weighted function (i.e., the reciprocal of the distance between a focal individual and its neighbors) because the former method cannot explain the variations in neighbor influence radius among species. Moreover, species of different neighbor influence radius have different Gaussian density neighbor kernel. The Gaussian weight function (*W*_*i*_) decreased with the spatial distance between focal seedling and its adult tree neighbors (Dist_*i*_), which indicates the neighbors that are more closely to focal individual plant will have stronger effects (Figure 3).

The neighbor influence radius depended on the specific species and its topographic position. Given the relative small variation in elevation (<6 m), we chose the valley position’ neighbor radius for these seven major species (Table S1, Yang, 2006), and the nearest phylogenetic‐related species’ neighbor influence radius for species that are not listed in Table S1. A phylogenetic tree was established using Phylomatic based on angiosperm phylogeny group (APG) III backbone phylogeny (http://phylodiversity.net/phylomatic/).

### 2.4 Abiotic variables

The abiotic variables included soil properties and topography. The 9.12‐ha plot was divided into square grids of 20 × 20 m. The elevation was measured at every grid point of 20 × 20 m squares. For soil properties, three soil samples were taken to a depth of 10 cm at random distance combinations of “0, 2, and 5 m”; “0, 2, and 8 m”; or “0, 5, and 8 m” in a random direction from grid points. In total, 780 soil samples were obtained for the 9.12‐ha plot (Figure 2). Ten soil properties were recorded as follows: organic carbon (C), total nitrogen (TN), hydrolyzable nitrogen (HN), total phosphorus (TP), available phosphorus (AP), rapidly available potassium (RAK), soil pH, volumetric moisture (VM), mass moisture (MM), and bulk density (BD). The values of abiotic variables measured at 20 × 20 m grid were interpolated to a 5 × 5 m grid with ordinary kriging, a popular geostatistical analysis tools. A principal components analysis (PCA) was performed to reduce the multicollinearity of soil variables. The first three principal components accounted for 71% of total variation in the ten soil variables. The first principal component (31% of the total variation) was associated with high C, TN, pH, and soil moisture, the second principal component (27% of the total variation) with low C and AP, and high TP and BD, the third principal component (13% of the total variation) with high HN and RAK (Table 1).

**Table 1:** Variable loadings for principal component analysis on soil properties

Soil variable	PC1	PC2	PC3
Organic carbon (C)	0.82	−0.48	−0.01
Total nitrogen (TN)	0.86	−0.27	0.15
Hydrolyzable nitrogen (HN)	0.32	0.18	0.67
Total phosphorus (TP)	−0.14	0.68	0
Available phosphorus (AP)	−0.01	−0.78	0.1
Rapidly available potassium (RAK)	−0.11	−0.3	0.84
pH	0.69	0.41	0.24
Volumetric moisture (VM)	0.48	0.64	0.22
Mass moisture (MS)	0.9	0.09	−0.02
Bulk density (BD)	−0.08	0.77	−0.08
Variation explained	31%	27%	13%

### 2.5 Data analysis

The survival of individual seedling from 2011 to 2015 was modeled as a function of biotic neighbors and abiotic habitat variables using the lme4 package in R software (R3.1.3; http://www.r-project.org) for generalized linear mixed models (GLMMs) with binominal distribution of errors (Bolker et al., 2009). The response variable was a logistic transformation of the seedling survival status, either 1 (alive) or 0 (dead). The seedling survival included seedling height as a covariate to account for age effects and two random variables, seedling plots to account for possible spatial correlation and species to account for species differences in their responses to neighborhood effects (Chen et al., 2010; Comita & Hubbell, 2009; Lin, Comita, Zheng, & Cao, 2012). The data of continuous explanatory variables were standardized prior to the statistical analysis.

To test the relative importance of biotic and abiotic variables, four candidate models were constructed: (1) a null model including seedling height as a only fixed effect; (2) a biotic model in which the fixed effects of seedling and adult tree neighbors were added to the null model; (3) an abiotic model in which the fixed effects of soil properties and topography were added to the null model; and (4) a full model in which all fixed effects of variables were added to the null model. Models were compared using Akaike's information criterion (AIC), and models with a AIC difference <2 were judged equally valid (Burnham & Anderson, 2002). The variance explained by fixed factors was included in marginal *R* ^2^ (*R* ^2^_mar_) and that by both fixed and random was in conditional *R* ^2^ (*R* ^2^_con_) of the models (Nakagawa, Schielzeth, & O'Hara, 2013).

Seedling survival was examined at three scales. First, the seedlings of all species were included with species and plot as random effects in the model. Second, species were included in the model by different functional groups of different growth forms (tree or shrub), species leaf habits (evergreen or deciduous), shade tolerance (shade‐tolerant or shade‐intolerant), with species and plot as random effects. Finally, the analysis of seedling survival was conducted by individual species for five most abundant tree species (*n* > 99 seedlings), including *Abies nephrolepis*,* Picea koraiensis*,* Acer ukurunduense*,* Pinus koraiensis,* and *Betula platyphylla*, with plot as a random effect.

## 3 RESULTS

**Figure 4:**
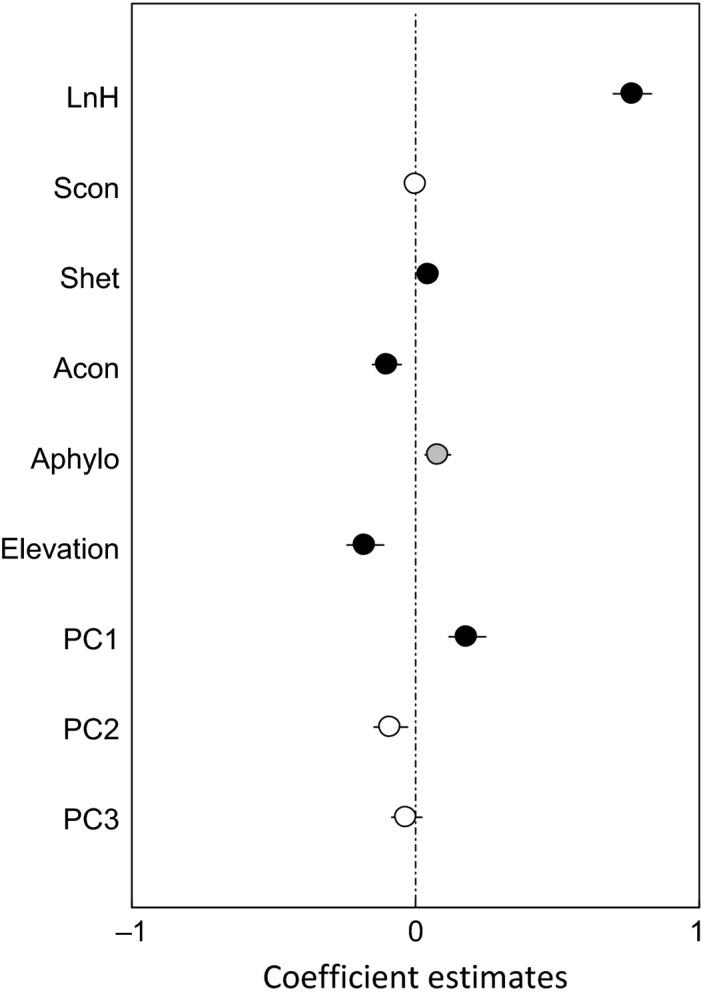
Estimated effects (mean ± *SE*) of variables on seedling survival by the best‐fit models of all species. Black circles represent for significant effects (*p *<* *.05), gray circles for marginally significant effects (.05 ≤ *p *<* *.1) and white circles for no significance

**Figure 5:**
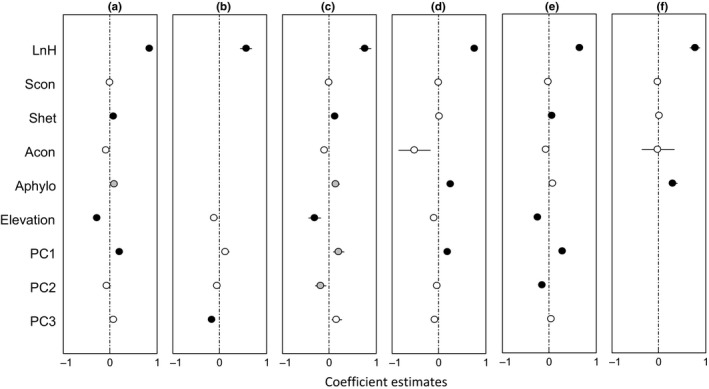
Estimated effects (mean ± *SE*) of variables on seedling survival by the best‐fit models of different functional groups, tree seedlings (a), shrub seedlings (b), evergreen seedlings (c), deciduous seedlings (d), shade‐tolerant seedlings (e), and shade‐intolerant seedlings (f). Black circles represent for significant effects (*p* < .05), gray circles for marginally significant effects (.05 ≤ *p *<* *.1), and white circles for no significance

**Figure 6:**
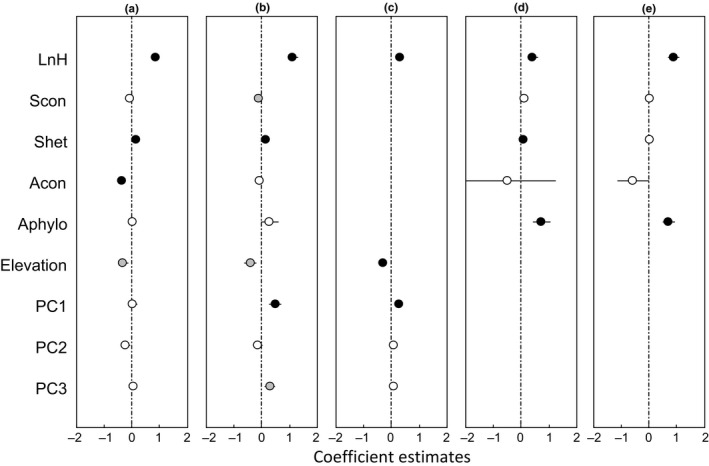
Estimated effects (mean ± *SE*) of variables on seedling survival by the best‐fit models of five individual species, *Abies nephrolepis* (a), *Picea koraiensis* (b), *Acer ukurunduense* (c), *Pinus koraiensis* (d), and *Betula platyphylla* (e). Black circles represent for significant effects (*p *<* *.05), gray circles for marginally significant effects (.05 ≤ *p *<* *.1), and white circles for no significance

Of the 6,256 tree and shrub seedlings tagged in 2011, 3,581, 57.24%, were still alive in 2015. The variables used in the models were summarized in Table 2. The probability of seedling survival generally increased with seedling height (Figures 4, 5, 6), and effects of other variables in the best‐fit models are described below.

**Table 2:** Summaries of variables in seedling survival analyses

Variables	Range	Mean	Median
Seedling effect (2 × 2 m)			
No. of conspecific (*S*_con_)	0–28	4.1961	3
No. of heterospecific (*S*_het_)	0–29	7.7043	7
Adult tree effect (dbh ≥ 1 cm)			
Conspecific basal area (*A*_con_)/m^2^	0–0.4584	0.0244	0.0003
Phylogenetic dissimilarity (*A*_phylo_)	0.0982–28.1179	2.5628	1.9626
Environmental properties			
Elevation	346.5345–352.3157	349.9558	350.011
Soil PC1	−2.5273–1.6913	0.1406	0.3993
Soil PC2	−3.4390–1.9085	−0.0254	0.2252
Soil PC3	−2.9517–2.5843	−0.0482	−0.0125

### 3.1 All‐species model

All biotic and abiotic factors were retained in the best‐fit seedling survival model for all species. The fixed factors (seedling height, biotic neighbors, and habitat variables) explained 11.4% of the variance (Tables 3 and S2). Both heterospecific seedling density and soil PC1 had significant, positive effects on seedling survival, as indicated by coefficient estimates (>0). In contrast, seedling survival decreased with conspecific adult tree density and elevation (Figure 4).

**Table 3:** Akaike's information criterion (AIC) and ΔAIC values of seedling survival models. The best‐fit models are highlighted in bold (see Figures 4, 5, 6 and S3 for coefficient estimates of the best‐fit models)

Level of modeling	Null		Biotic		Abiotic		Full	
	AIC	ΔAIC	AIC	ΔAIC	AIC	ΔAIC	AIC	ΔAIC
All	7,469.59	50.24	7,445.72	26.37	7,440.32	20.96	**7,419.36**	**0**
By functional level								
Growth form								
Tree	4,075.68	64.62	4,041.74	30.68	4,046.23	35.17	**4,011.06**	**0**
Shrub	3,466.78	11.56	**3,455.22**	**0**	3,458.97	3.75	3,459.72	4.50
Leaf habit								
Evergreen	2,435.35	57.46	2,394.50	16.60	2,418.23	40.34	**2,377.89**	**0**
Deciduous	5,047.34	31.76	5,029.98	14.40	5,029.31	13.73	**5,015.58**	**0**
Shade tolerance								
Shade‐tolerant	4,933.18	67.23	4,907.05	41.10	4,891.05	25.10	**4,865.95**	**0**
Shade‐intolerant	2,574.86	6.96	**2,567.90**	**0**	2,576.79	8.89	2,570.98	3.08
By individual species level								
*Abies nephrolepis*	1,083.10	47.74	**1,035.51**	**0.16**	1,072.37	37.02	**1,035.36**	**0**
*Picea koraiensis*	875.52	23.64	861.54	9.66	870.08	18.20	**851.88**	**0**
*Acer ukurunduense*	651.93	23.20	654.31	25.58	**628.73**	**0**	**630.31**	**1.58**
*Pinus koraiensis*	513.41	9.12	**504.29**	**0**	519.82	15.53	510.24	5.95
*Betula platyphylla*	416.51	9.66	**406.84**	**0**	418.36	11.51	408.57	1.73

### 3.2 Functional group models

#### 3.2.1 Growth form

Again, the best‐fit model for trees was the full model, with fixed factors (seedling height, biotic neighbors, and habitat variables) explaining 14% of the total variance. The seedling survival was significantly increased with heterospecific seedling density and soil PC1, and marginally increased with heterospecific phylogenetic dissimilarity, but decreased with elevation. In comparison, the survival of shrub species was best described by the abiotic model, with fixed factors (seedling height and habitat variables) explaining 4.8% of the total variance. The seedling survival of shrubs decreased with soil PC3 that was primarily associated with HN and RAK (Tables 3 and S2, Figure 5a,b).

#### 3.2.2 Leaf habit

The best survival model by leaf habits was the full model, with the fixed factors (seedling height, biotic neighbors and habitat variables) explaining 14.8% of the total variance for evergreen seedlings and 11.3% for deciduous seedling (Tables 3 and S2). A positive effect of heterospecific phylogenetic dissimilarity on seedling survival was stronger for deciduous seedlings than for evergreen seedlings. The model for evergreen seedlings included positive effects of heterospecific seedling density and soil PC1, but negative effects of elevation and soil PC2. Comparatively, the survival of deciduous seedlings increased with higher soil PC1 (Figure 5c,d).

#### 3.2.3 Shade tolerance

The best‐fit model for shade‐tolerant seedlings was the full model, whereas that for shade‐intolerant seedlings was the biotic model. The fixed factors (seedling height, biotic neighbors, and habitat variables) explained 12.7% of the total variance for shade‐tolerant seedlings and 10.7% for shade‐intolerant seedlings (Tables 3 and S2). For shade‐tolerant seedlings, their survival increased with heterospecific seedling density and soil PC1, but decreased with increasing elevation and soil PC2. For shade‐intolerant seedlings, their survival significantly increased only with heterospecific phylogenetic dissimilarity (Figure 5).

### 3.3 Individual species model

The best‐fit model for *A. nephrolepis* and *P. koraiensis* was the full model that explained 22.5% and 20.1% of the total variance by the fixed factors (seedling height, biotic neighbors, and habitat variables). In comparison, the best‐fit model for *A. ukurunduense* was the abiotic model, explaining 10.8% of the total variance by fixed factors (habitat variables) and that for *P. koraiensis* and *B. platyphylla* was the biotic model explaining 9.5% and 18.7% of the total variance, respectively, by the fixed factors (Tables 3 and S2). The seedling survival of *A. nephrolepis* species increased with heterospecific seedling density, conspecific adult tree density, and elevation (Figure 6a), while that, for *P. koraiensis* decreased with conspecific seedling density and elevation, and increased with soil PC1, heterospecific seedling density, and soil PC3 (Figure 6b). The survival of *A. ukurunduense* was significantly affected by soil PC1 (on the positive side) and elevation (on the negative side), but not by biotic factors (Figure 6c). The survival probability of *P. koraiensis* and *B. platyphylla* seedlings increased with heterospecific phylogenetic dissimilarity (Figure 6d,e).

## 4 DISCUSSION

### 4.1 Effects of density, habitat, and phylogenetic dissimilarity

At all levels of survival analyses, seedling height was positively associated with seedling survival, consistent with the findings by others (Metz et al., 2010). The possible reason is that large seedlings are likely less susceptible to biotic stresses (e.g., herbivores and pathogens) and in better positions in competition for light (Chen et al., 2010; Comita & Hubbell, 2009; Queenborough et al., 2007).

Numerous studies have indicated that NDD is an important mechanism for maintaining species coexistence at seedling stage (Bagchi, Press, & Scholes, 2010; Johnson et al., 2014; Nathan & Muller‐Landau, 2000; Yan, Zhang, Wang, Zhao, & von Gadow, 2015). This is also true in our study with a strong CNDD effect on seedling survival (Figures 4 and 6a). This is likely due to the fact that conspecific adult trees have similar resource requirements, as well as share similar pests and pathogens with seedlings, which is conductive to high competition and mortality. On the other hand, seedling survival was not negatively affected by conspecific seedling density, which is similar to the findings by Paine, Harms, Schnitzer, and Carson (2008) and Svenning, Fabbro, and Wright (2008), but different from those by Chen et al. (2010) and Comita and Hubbell (2009). This inconsistency may result from the levels of competition among seedlings; the seedling of small size and low density would not be strongly influenced by CNDD (Paine et al., 2008; Svenning et al., 2008). As expected, seedling survival was positively associated with heterospecific seedling density (Figures 4, 5a,c,e, and 6a,b,d), an indication of heterospecific positive density dependence (HPDD). As suggested by the “species herd protection hypothesis” (Peters, 2003), the seedlings of more heterospecific neighbors would have less competition and therefore higher chance of survival.

Our results showed that heterospecific phylogenetic dissimilarity was positively associated with seedling survival, indicating positive effects of PNDD in the spruce‐fir valley forest, northeastern China (Figures 4, 5a,c,d,f, and 6d,e). Focal seedlings would have a low survival when surrounded by conspecific tree neighbors, due to increased competition for resources and sharing of common pests and pathogens (Gilbert & Webb, 2007; Liu et al., 2012; Novotny et al., 2002). On the other hand, conspecific tree neighbors could also be positively associated with seedling survival (Lebrija‐Trejos, Wright, Hernández, & Reich, 2014; Lu et al., 2015; Zhu et al., 2015), possibly through habitat affinities. These different effects of phylogenetic neighbors reflect the variation of phylogenetic niche conservatism with site conditions.

Contrary to some observations showing limited effects of abiotic factors on seedling survival (Lu et al., 2015; Shibata et al., 2010; Wang et al., 2012), abiotic factors such as high soil moisture, pH, soil total carbon, and soil total nitrogen were all positively associated with seedling survival in the spruce‐fir valley forest, northeastern China. The possible reasons would be that seedlings have shallow roots and therefore limited access to resources, and should benefit from high moisture, pH, carbon, and nitrogen in the soil (Engelbrecht, Kursar, & Tyree, 2005; Lin et al., 2012). This may also be applied to variations in topography that the increase in elevation was associated with decreased total carbon, total nitrogen, and moisture content in the soil (*p *<* *0.001), leading to decrease in seedling survival (Figures 4, 5a,c,e, and 6c). The strong influences of abiotic factors suggest that the seedlings in the spruce‐fir valley forest are restricted by soil moisture and nutrients and very sensitive to changes of resources availability, even by slight variation in elevation (<6 m).

Small proportions of the total variance (12.6%–58.5%) were accounted for by the selected fixed and random variables, indicating significant influences of other factors, such as pests, diseases, forest structure, species functional traits, and microclimatic conditions on seedling survival. Future studies should examine the influences of these factors, particularly under the context of climate change. In this study, random effects accounted for more variance than fixed effects (Table S2), indicating significant differences in seedling survival among species and spatial variations of site conditions.

### 4.2 Differences among functional groups

Our results do not support the suggestions that trees produce more seeds than shrubs and are likely to affected more by CNDD (King, Wright, & Connell, 2005; Terborgh & Petren, 1991; Terborgh et al., 2014). Comparatively, tree seedlings were more influenced by biotic adult tree neighbors than shrubs, indicating greater needs for resource sharing and pressures for competition. Between evergreen and deciduous species, deciduous seedlings uptake and allocate more resources on growth, whereas evergreen seedlings tend to allocate more on defensive compounds, which would help survival (Coley & Barone, 1996; Villar, Robleto, De Jong, & Poorter, 2006). The greater growth by deciduous seedlings would require a greater level of resource sharing and therefore are more influenced by PNDD. We did not see greater influences of CNDD on survival of shade‐intolerant seedlings due to their low carbohydrates and tissue density, and high vulnerability to competition and external damages (Piao, Chun, Yang, & Cheon, 2014); in facts, the survival of shade‐intolerant seedlings was not affected by conspecific neighbor density. On the other hand, the survival of shade‐intolerant seedlings decreased with the amount of phylogenetic‐related neighbors, indicating greater importance of PNDD than CNDD in survival of shade‐intolerant seedlings.

### 4.3 Differences among individual species

Different species have difference physiological and functional traits and therefore different critical factors for seedling survival. In our study, the survival of *A. nephrolepis* seedlings was negatively correlated with conspecific adult tree density, while that of *P. koraiensis* seedlings was negatively with conspecific seedling density (Figure 6a,b). The CNDD influences on seedling survival, however, disappeared in the all‐species model that excluded *A. nephrolepis* and *P. koraiensis* indicating that the CNDD effects were mainly on the two species (Figure S3). Their survival was affected by both biotic neighbors and abiotic factors according to the best‐fit models. Comparatively, the survival of *A. ukurunduense* depended on abiotic factors such as high soil nutrient contents and moisture and therefore niche partitioning, whereas heterospecific phylogenetic dissimilarity was critical to the survival of a *P. koraiensis* and *B. platyphylla*.

## 5 CONCLUSION

The seedling survival in a spruce‐fir valley forest, northeastern China, depended on both biotic neighbors and habitat heterogeneity (elevation and soil properties). However, the relative importance of these factors varied by species and functional groups. Conspecific negative density dependence (CNDD) was important to the survival of dominant species including *A. nephrolepis* and *P. koraiensis*, whereas PNDD was critical to the survival of *P. koraiensis* and *B. platyphylla*, as well as to the species groups of tree seedlings, deciduous seedlings, or shade‐intolerant seedlings. For shrubs and *A. ukurunduense*, habitat heterogeneity was significant to their seedling survival. In this study, all the studied variables only explained small proportions of the total variance, suggesting the needs to examine the influences of other factors such as pests, diseases, herbivores, forest structure, species functional traits, and microclimatic conditions on seedling survival in the future.

## CONFLICT OF INTEREST

None declared.

## References

[ece33030-bib-0001] Bagchi, R. , Press, M. C. , & Scholes, J. D. (2010). Evolutionary history and distance dependence control survival of dipterocarp seedlings. Ecology Letters, 13, 51–59. 19849708 10.1111/j.1461-0248.2009.01397.x

[ece33030-bib-0002] Bai, X. , Queenborough, S. A. , Wang, X. , Zhang, J. , Li, B. , Yuan, Z. , … Hao, Z. (2012). Effects of local biotic neighbors and habitat heterogeneity on tree and shrub seedling survival in an old‐growth temperate forest. Oecologia, 170, 755–765. 22644047 10.1007/s00442-012-2348-2

[ece33030-bib-0003] Bolker, B. M. , Brooks, M. E. , Clark, C. J. , Geange, S. W. , Poulsen, J. R. , Stevens, M. H. H. , & White, J. S. S. (2009). Generalized linear mixed models: A practical guide for ecology and evolution. Trends in Ecology & Evolution, 24, 127–135. 19185386 10.1016/j.tree.2008.10.008

[ece33030-bib-0004] Burnham, K. P. , & Anderson, D. R. (2002). Model selection and multimodel inference: A practical information‐theoretic approach, 2nd ed. New York, NY: Springer.

[ece33030-bib-0006] Chen, L. , Mi, X. , Comita, L. S. , Zhang, L. , Ren, H. , & Ma, K. (2010). Community‐level consequences of density dependence and habitat association in a subtropical broad‐leaved forest. Ecology Letters, 13, 695–704. 20412278 10.1111/j.1461-0248.2010.01468.x

[ece33030-bib-0007] Chen, Y. , Wright, S. J. , Muller‐Landau, H. C. , Hubbell, S. P. , Wang, Y. , & Yu, S. (2016). Positive effects of neighborhood complementarity on tree growth in a Neotropical forest. Ecology, 97, 776–785. 27197403 10.1890/15-0625.1

[ece33030-bib-0008] Chesson, P. (2000). Mechanisms of maintenance of species diversity. Annual Review of Ecology and Systematics, 31, 343–366.

[ece33030-bib-0009] Clark, J. S. , Macklin, E. , & Wood, L. (1998). Stages and spatial scales of recruitment limitation in southern Appalachian forests. Ecological Monographs, 68, 213–235.

[ece33030-bib-0010] Coley, P. D. , & Barone, J. A. (1996). Herbivory and plant defenses in tropical forests. Annual Review of Ecology and Systematics, 27, 305–335.

[ece33030-bib-0011] Comita, L. S. , & Hubbell, S. P. (2009). Local neighborhood and species’ shade tolerance influence survival in a diverse seedling bank. Ecology, 90, 328–334. 19323215 10.1890/08-0451.1

[ece33030-bib-0012] Comita, L. S. , Muller‐Landau, H. C. , Aguilar, S. , & Hubbell, S. P. (2010). Asymmetric density dependence shapes species abundances in a tropical tree community. Science, 329, 330–332. 20576853 10.1126/science.1190772

[ece33030-bib-0013] Comita, L. S. , Queenborough, S. A. , Murphy, S. J. , Eck, J. L. , Xu, K. , Krishnadas, M. , … Zhu, Y. (2014). Testing predictions of the Janzen‐Connell hypothesis: A meta‐analysis of experimental evidence for distance‐ and density‐dependent seed and seedling survival. Journal of Ecology, 102, 845–856. 25253908 10.1111/1365-2745.12232 PMC4140603

[ece33030-bib-0014] Engelbrecht, B. M. J. , Kursar, T. A. , & Tyree, M. T. (2005). Drought effects on seedling survival in a tropical moist forest. Trees, 19, 312–321.

[ece33030-bib-0016] Gilbert, G. S. , & Webb, C. O. (2007). Phylogenetic signal in plant pathogen‐host range. Proceedings of the National Academy of Sciences, 104, 4979–4983. 10.1073/pnas.0607968104 PMC1829250 17360396

[ece33030-bib-0017] Jansen, P. A. , Visser, M. D. , Wright, S. J. , Rutten, G. , Muller‐Landau, H. C. , & Rejmanek, M. (2014). Negative density dependence of seed dispersal and seedling recruitment in a Neotropical palm. Ecology Letters, 17, 1111–1120. 25039608 10.1111/ele.12317

[ece33030-bib-0018] Janzen, D. H. (1970). Herbivores and the number of tree species in tropical forests. The American Naturalist, 104, 501–528.

[ece33030-bib-0019] Johnson, D. J. , Beaulieu, W. T. , Bever, J. D. , & Clay, K. (2012). Conspecific negative density dependence and forest diversity. Science, 336, 904–907. 22605774 10.1126/science.1220269

[ece33030-bib-0020] Johnson, D. J. , Bourg, N. A. , Howe, R. , Mcshea, W. J. , Wolf, A. , & Clay, K. (2014). Conspecific negative density‐dependent mortality and the structure of temperate forests. Ecology, 95, 2493–2503.

[ece33030-bib-0021] King, D. A. , Wright, S. J. , & Connell, J. H. (2005). The contribution of interspecific variation in maximum tree height to tropical and temperate diversity. Journal of Tropical Ecology, 22, 11–24.

[ece33030-bib-0022] Kitajima, K. , & Poorter, L. (2010). Tissue‐level leaf toughness, but not lamina thickness, predicts sapling leaf lifespan and shade tolerance of tropical tree species. New Phytologist, 186, 708–721. 20298481 10.1111/j.1469-8137.2010.03212.x

[ece33030-bib-0023] Kobe, R. (1997). Carbohydrate allocation to storage as a basis of interspecific variation in sapling survivorship and growth. Oikos, 80, 226–233.

[ece33030-bib-0024] Lebrija‐Trejos, E. , Wright, S. J. , Hernández, A. , & Reich, P. B. (2014). Does relatedness matter? Phylogenetic density‐dependent survival of seedlings in a tropical forest. Ecology, 95, 940–951. 24933813 10.1890/13-0623.1

[ece33030-bib-0025] Lin, L. , Comita, L. S. , Zheng, Z. , & Cao, M. (2012). Seasonal differentiation in density‐dependent seedling survival in a tropical rain forest. Journal of Ecology, 100, 905–914.

[ece33030-bib-0026] Liu, X. , Liang, M. , Etienne, R. S. , Wang, Y. , Staehelin, C. , & Yu, S. (2012). Experimental evidence for a phylogenetic Janzen‐Connell effect in a subtropical forest. Ecology Letters, 15, 111–118. 22082078 10.1111/j.1461-0248.2011.01715.x

[ece33030-bib-0027] Lu, J. , Johnson, D. J. , Qiao, X. , Lu, Z. , Wang, Q. , & Jiang, M. (2015). Density dependence and habitat preference shape seedling survival in a subtropical forest in central China. Journal of Plant Ecology, 8, 568–577.

[ece33030-bib-0029] Metz, M. R. (2012). Does habitat specialization by seedlings contribute to the high diversity of a lowland rain forest? Journal of Ecology, 100, 969–979.

[ece33030-bib-0030] Metz, M. R. , Sousa, W. P. , & Renato, V. (2010). Widespread density‐dependent seedling mortality promotes species coexistence in a highly diverse Amazonian rain forest. Ecology, 91, 3675–3685. 21302838 10.1890/08-2323.1

[ece33030-bib-0031] Myers, J. A. , & Kitajima, K. (2007). Carbohydrate storage enhances seedling shade and stress tolerance in a neotropical forest. Journal of Ecology, 95, 383–395.

[ece33030-bib-0032] Nakagawa, S. , Schielzeth, H. , & O'Hara, R. B. (2013). A general and simple method for obtaining *R* ^2^ from generalized linear mixed‐effects models. Methods in Ecology and Evolution, 4, 133–142.

[ece33030-bib-0033] Nathan, R. , & Muller‐Landau, H. C. (2000). Spatial patterns of seed dispersal, their determinants and consequences for recruitment. Trends in Ecology & Evolution, 15, 278–285. 10856948 10.1016/s0169-5347(00)01874-7

[ece33030-bib-0034] Novotny, V. , Basset, Y. , Miller, S. E. , Weiblen, G. D. , Bremer, B. , Cizek, L. , & Drozd, P. (2002). Low host specificity of herbivorous insects in a tropical forest. Nature, 416, 841–844. 11976681 10.1038/416841a

[ece33030-bib-0035] Paine, C. E. T. , Harms, K. E. , Schnitzer, S. A. , & Carson, W. P. (2008). Weak competition among tropical tree seedlings: Implications for species coexistence. Biotropica, 40, 432–440.

[ece33030-bib-0036] Paine, C. E. , Norden, N. , Chave, J. , Forget, P. M. , Fortunel, C. , Dexter, K. G. , & Baraloto, C. (2012). Phylogenetic density dependence and environmental filtering predict seedling mortality in a tropical forest. Ecology Letters, 15, 34–41. 22004454 10.1111/j.1461-0248.2011.01705.x

[ece33030-bib-0037] Peters, H. A. (2003). Neighbour‐regulated mortality: The influence of positive and negative density dependence on tree populations in species‐rich tropical forests. Ecology Letters, 6, 757–765.

[ece33030-bib-0038] Piao, T. , Chun, J. H. , Yang, H. M. , & Cheon, K. (2014). Negative density dependence regulates two tree species at later life stage in a temperate forest. PLoS One, 9, e103344. 25058660 10.1371/journal.pone.0103344 PMC4110017

[ece33030-bib-0039] Piao, T. , Comita, L. S. , Jin, G. , & Kim, J. H. (2013). Density dependence across multiple life stages in a temperate old‐growth forest of northeast China. Oecologia, 172, 207–217. 23053238 10.1007/s00442-012-2481-y PMC3627022

[ece33030-bib-0040] Queenborough, S. A. , Burslem, D. F. R. P. , Garwood, N. C. , & Valencia, R. (2007). Neighborhood and community interactions determine the spatial pattern of tropical tree seedling survival. Ecology, 88, 2248–2258. 17918403 10.1890/06-0737.1

[ece33030-bib-0041] Queenborough, S. A. , Burslem, D. F. R. P. , Garwood, N. C. , & Valencia, R. (2009). Taxonomic scale‐dependence of habitat niche partitioning and biotic neighbourhood on survival of tropical tree seedlings. Proceedings of the Royal Society B, 276, 4197–4205. 19740886 10.1098/rspb.2009.0921 PMC2821336

[ece33030-bib-0042] Shi, B. , & Jin, G. (2016). Variability of soil respiration at different spatial scales in temperate forests. Biology and Fertility of Soils, 52, 561–571.

[ece33030-bib-0043] Shibata, M. , Masaki, T. , Tanaka, H. , Niiyama, K. , Iida, S. , Abe, S. , & Nakashizuka, T. (2010). Effects of abiotic and biotic factors and stochasticity on tree regeneration in a temperate forest community. Ecoscience, 17, 137–145.

[ece33030-bib-0044] Svenning, J. C. , Fabbro, T. , & Wright, S. J. (2008). Seedling interactions in a tropical forest in Panama. Oecologia, 155, 143–150. 17965886 10.1007/s00442-007-0884-y

[ece33030-bib-0045] Terborgh, J. , & Petren, K. (1991). Development of habitat structure through succession in an Amazonian floodplain forest. Netherlands: Springer.

[ece33030-bib-0046] Terborgh, J. , Zhu, K. , Alvarez‐Loayza, P. , & Cornejo‐Valverde, F. (2014). How many seeds does it take to make a sapling? Ecology, 95, 991–999. 24933817 10.1890/13-0764.1

[ece33030-bib-0047] Uriarte, M. , Condit, R. , Canham, C. D. , & Hubbell, S. P. (2004). A spatially explicit model of sapling growth in a tropical forest: Does the identity of neighbours matter? Journal of Ecology, 92, 348–360.

[ece33030-bib-0049] Villar, R. , Robleto, J. R. , De Jong, Y. , & Poorter, H. (2006). Differences in construction costs and chemical composition between deciduous and evergreen woody species are small as compared to differences among families. Plant, Cell and Environment, 29, 1629–1643. 10.1111/j.1365-3040.2006.01540.x 16898023

[ece33030-bib-0050] Wang, X. , Comita, L. S. , Hao, Z. , Davies, S. J. , Ye, J. , Lin, F. , & Yuan, Z. (2012). Local‐scale drivers of tree survival in a temperate forest. PLoS One, 7, e29469. 22347996 10.1371/journal.pone.0029469 PMC3278403

[ece33030-bib-0051] Webb, C. O. , Gilbert, G. S. , & Donoghue, M. J. (2006). Phylodiversity‐dependent seedling mortality, size structure, and disease in a Bornean rain forest. Ecology, 87, S123–S131. 16922308 10.1890/0012-9658(2006)87[123:psmssa]2.0.co;2

[ece33030-bib-0052] Wright, J. S. (2002). Plant diversity in tropical forests: A review of mechanisms of species coexistence. Oecologia, 130, 1–14. 28547014 10.1007/s004420100809

[ece33030-bib-0053] Wu, J. , Swenson, N. G. , Brown, C. , Zhang, C. , Yang, J. , Ci, X. , & Lin, L. (2016). How does habitat filtering affect the detection of conspecific and phylogenetic density dependence? Ecology, 97, 1182–1193. 27349095 10.1890/14-2465.1

[ece33030-bib-0054] Yan, Y. , Zhang, C. , Wang, Y. , Zhao, X. , & von Gadow, K. (2015). Drivers of seedling survival in a temperate forest and their relative importance at three stages of succession. Ecology and Evolution, 5, 4287–4299. 26664679 10.1002/ece3.1688 PMC4667830

[ece33030-bib-0055] Yang, G. (2006). Study on the neighborhood influence radius of partial main tree species in northeastern China, Master Thesis, Northeast Forestry University (China).

[ece33030-bib-0056] Zhang, L. , Mi, X. , Shao, H. , & Ma, K. (2011). Strong plant‐soil associations in a heterogeneous subtropical broad‐leaved forest. Plant and Soil, 347, 211–220.

[ece33030-bib-0057] Zhu, Y. , Comita, L. S. , Hubbell, S. P. , Ma, K. , & Shefferson, R. (2015). Conspecific and phylogenetic density‐dependent survival differs across life stages in a tropical forest. Journal of Ecology, 103, 957–966.

[ece33030-bib-0058] Zhu, Y. , Mi, X. , Ren, H. , & Ma, K. (2010). Density dependence is prevalent in a heterogeneous subtropical forest. Oikos, 119, 109–119.

