# Supplementary material for: Effects of local biotic neighbors and habitat heterogeneity on seedling survival in a spruce‐fir valley forest, northeastern China
Source: Ecol Evol. 2017 May 18;7(13):4582–91. doi: 10.1002/ece3.3030 (PMC5496565; doi:10.1002/ece3.3030)
Supplement: Supplementary file 1 [file ECE3-7-4582-s001.docx]

**Appendix S1.** Neighbour influence radii (*R*) of major species in eastern mountains of northeastern China. The topographic position and radii used in this study are highlighted in bold.

| Species | Topographic position | | |
| --- | --- | --- | --- |
|  | Ridge | Slope | **Valley** |
| *Abies nephrolepis* | 5 | 7 | **6** |
| *Betula costata* | 6 | 5 | **5** |
| *Betula platyphylla* | 6 | 5 | **5** |
| *Picea koraiensis* | 7 | 6 | **6** |
| *Pinus koraiensis* | 6 | 5 | **6** |
| *Juglans mandshurica* | 5 | 5 | **5** |
| *Fraxinus mandschurica* | 6 | 6 | **7** |

**Appendix S2.** Model variance explained by fixed effects (*R^2^*_mar_) and total effects of fixed effects and random effects (*R^2^*_con_).

| Level of modelling | Null | |  | Biotic | |  | Abiotic | |  | Full | |
| --- | --- | --- | --- | --- | --- | --- | --- | --- | --- | --- | --- |
|  | *R^2^*_mar_(%) | *R^2^*_con_(%) |  | *R^2^*_mar_(%) | *R^2^*_con_(%) |  | *R^2^*_mar_(%) | *R^2^*_con_(%) |  | *R^2^*_mar_(%) | *R^2^*_con_(%) |
| All | 9.3 | 44.3 |  | 10.0 | 44.3 |  | 10.7 | 44.4 |  | 11.4 | 44.5 |
| By functional groups level |  |  |  |  |  |  |  |  |  |  |  |
| Growth form |  |  |  |  |  |  |  |  |  |  |  |
| Tree | 9.1 | 48.3 |  | 11.3 | 48.5 |  | 11.7 | 48.3 |  | 14.0 | 48.7 |
| Shrub | 3.6 | 35.0 |  | 4.8 | 34.2 |  | 4.2 | 35.0 |  | 5.4 | 34.4 |
| Leaf habit |  |  |  |  |  |  |  |  |  |  |  |
| Evergreen | 6.1 | 50.9 |  | 11.6 | 49.3 |  | 9.4 | 51.8 |  | 14.8 | 50.7 |
| Deciduous | 9.7 | 37.5 |  | 10.7 | 36.9 |  | 10.5 | 37.6 |  | 11.3 | 37.0 |
| Shade-tolerance |  |  |  |  |  |  |  |  |  |  |  |
| Shade-tolerant | 7.5 | 39.7 |  | 9.4 | 39.7 |  | 10.6 | 39.7 |  | 12.7 | 39.9 |
| Shade-intolerant | 9.7 | 41.6 |  | 10.7 | 41.8 |  | 10.2 | 41.8 |  | 11.2 | 42.0 |
| By individual species level |  |  |  |  |  |  |  |  |  |  |  |
| *Abies nephrolepis* | 8.5 | 55.4 |  | 20.7 | 54.0 |  | 13.0 | 56.9 |  | 22.5 | 55.0 |
| *Picea koraiensis* | 8.9 | 54.6 |  | 15.2 | 57.3 |  | 12.5 | 55.2 |  | 20.1 | 58.5 |
| *Acer ukurunduense* | 2.5 | 13.4 |  | 4.3 | 14.7 |  | 10.8 | 12.6 |  | 12.6 | 13.7 |
| *Pinus koraiensis* | 1.4 | 42.6 |  | 9.5 | 44.1 |  | 2.1 | 43.8 |  | 10.4 | 45.0 |
| *Betula platyphylla* | 10.1 | 28.0 |  | 18.7 | 32.8 |  | 13.0 | 27.8 |  | 21.3 | 30.5 |
|  |  |  |  |  |  |  |  |  |  |  |  |

**Appendix S3.** Estimated effects (mean ± SE) of variables on seedling survival by the best-fit model of all species, except for *Abies nephrolepis* and *Picea koraiensis*. Black circles represent for significant effects (*P* < 0.05), grey circles for marginally significant effects (0.05 ≤ *P* < 0.1) and white circles for no significance.
